# Do Transgenerational Epigenetic Inheritance and Immune System Development Share Common Epigenetic Processes?

**DOI:** 10.3390/jdb9020020

**Published:** 2021-05-12

**Authors:** Rwik Sen, Christopher Barnes

**Affiliations:** Active Motif, Incorporated, 1914 Palomar Oaks Way, Suite 150, Carlsbad, CA 92008, USA; cbarnes@activemotif.com

**Keywords:** transgenerational, epigenetic, development, immune system, chromatin, histone, non-coding RNA, prion

## Abstract

Epigenetic modifications regulate gene expression for development, immune response, disease, and other processes. A major role of epigenetics is to control the dynamics of chromatin structure, i.e., the condensed packaging of DNA around histone proteins in eukaryotic nuclei. Key epigenetic factors include enzymes for histone modifications and DNA methylation, non-coding RNAs, and prions. Epigenetic modifications are heritable but during embryonic development, most parental epigenetic marks are erased and reset. Interestingly, some epigenetic modifications, that may be resulting from immune response to stimuli, can escape remodeling and transmit to subsequent generations who are not exposed to those stimuli. This phenomenon is called transgenerational epigenetic inheritance if the epigenetic phenotype persists beyond the third generation in female germlines and second generation in male germlines. Although its primary function is likely immune response for survival, its role in the development and functioning of the immune system is not extensively explored, despite studies reporting transgenerational inheritance of stress-induced epigenetic modifications resulting in immune disorders. Hence, this review draws from studies on transgenerational epigenetic inheritance, immune system development and function, high-throughput epigenetics tools to study those phenomena, and relevant clinical trials, to focus on their significance and deeper understanding for future research, therapeutic developments, and various applications.

## 1. Introduction

Several mechanisms of epigenetic inheritance have been reported [1]. At the transcriptional level, the mechanisms are regulated by DNA methylation, histone modifications, and transcription factors. Mechanisms at the RNA level are RNA splicing and RNA-mediated post-transcriptional silencing. At the protein levels, mechanisms include organellar translation, protein truncation and folding, post-translational chemical modifications, and homologous and non-homologous protein interactions. However, not all epigenetic inheritance patterns have yet been identified to be retained across several generations; those which persist are referred to as transgenerational epigenetic inheritance.

Precisely, transgenerational epigenetic inheritance for female germlines refers to phenotypes that arise in the F0 generation in response to stimuli and continue to be transmitted at least up to F3, despite generations after F0 not being subjected to the stimuli. Similar modes of transmission at least up to F2 in male germlines are considered transgenerational. The generation numbers of three and two are determined for females and males, respectively, because of the exposure mode of the progeny to stimuli.

It is important to learn about transgenerational epigenetic inheritance as the process has been detected in eukaryotes across the spectrum from yeast to humans. Furthermore, transgenerational epigenetic inheritance is reported at the level of chromatin structure, DNA methylation, histone modifications, transcription, translation, and protein folding. It is important to understand why, despite getting most parental epigenetic marks erased, some epigenetic marks persist in a developing embryo to be transmitted across multiple generations. In this direction, a major function of transgenerational epigenetic inheritance is proposed to be fitness for adaptability and survival, manifested through immune response to unfavorable environments or stimuli, as seen from studies on several species.

Despite the above observations and considerable information on general epigenetic regulation of immune system development and function, the complete role of transgenerational epigenetic inheritance in this context is not well-explored. Hence, this review focuses on studies that address various aspects of the above context including epigenetic mechanisms that undergo transgenerational inheritance with pathological outcomes, and impact immune system development and function.

Findings from diverse studies have the potential to inform future research on why and how specific epigenetic modifications undergo transgenerational inheritance, and how they affect the immune system. The results can possibly impact a wide range of applications including therapies against various diseases, addressing issues like substance abuse and mental health, and benefitting agriculture, the environment, and ecology, as discussed in subsequent sections.

## 2. Overview of Epigenetics

The development of the embryo is tightly orchestrated by various epigenetic mechanisms caused by multiple factors, like gene-environment interaction, and modifications of chromatin, histones, DNA, RNA, and proteins. Inside a eukaryotic nucleus, DNA wraps around histone proteins for compact packaging into a condensed chromatin structure. In the chromatin, histones exist as octamers comprised of two copies each of core histones H2A, H2B, H3, and H4, along with linker histone H1. From a structural perspective, 147 bp of DNA wraps around one histone octamer to form a nucleosome, which is the fundamental subunit of chromatin.

Condensed packaging of DNA into chromatin structure is called heterochromatin, which renders DNA inaccessible to transcription factors. On the other hand, loose DNA packaging, called euchromatin, makes DNA accessible to transcription factors. Hence, euchromatin is indicative of transcription activation, while heterochromatin corresponds to transcription repression. Such remodeling of chromatin is ATP-dependent and is caused by factors called chromatin remodelers. Histone modifications and methylation of specific residues on DNA regulate chromatin dynamics and hence, gene expression. Accordingly, chromatin remodelers and enzymes regulating histone modifications and DNA methylation exert epigenetic regulation at the level of transcription.

Disruption of epigenetic regulation leads to diseases like cancers, neurodegeneration, and developmental disorders. Hence, it is important to understand how epigenetic information is processed, stored, and transmitted during the lifecycle of an organism in the context of development and disease. Interestingly, although germ cells carry epigenetic information, the developing embryo undergoes epigenetic erasing and resetting of epigenetic marks. However, some of the parental epigenetic marks are retained, and a subset of those are preserved across multiple generations, which establishes the field of transgenerational epigenetic inheritance.

## 3. Transgenerational Epigenetic Inheritance

Epigenetic alterations can be induced randomly and from a myriad of environmental factors including toxins, nutrition, and stress. If the epigenetic change is induced in a gestating female (F0 generation), both the fetus in utero (F1 generation) and the germline of the fetus (F2 generation) are considered to be directly exposed, therefore, making the F3 generation the first instance of transgenerational inheritance [2,3].

In contrast, if the epigenetic change is induced in a male, then only he (F0) and his germline (F1) are considered to be directly exposed, making the F2 generation the first instance of transgenerational inheritance [2,3]. As such, transgenerational effects are those phenotypes that are inherited in the third generation from an original organism in the case of females, and inherited in the second generation for males [2,3,4,5,6].

The phenotype caused by transgenerational epigenetic inheritance results from a direct impact on the original F0 organism, and is inherited in a non-DNA-based mechanism. Any effect spanning a generational timescale that is less than F3 in females and F2 in males is called parental or intergenerational. However, mechanisms between many transgenerational and intergenerational effects overlap [2]. In an excellent review, Nilsson et al. define transgenerational epigenetic inheritance as “germline mediated inheritance of epigenetic information between generations in the absence of continued direct environmental influences” [7].

### 3.1. Transgenerational Epigenetic Inheritance in Development

There are two main developmental periods: during the early embryonic stage post-fertilization and during the specification of germ cell at gonadal sex determination [8,9]. During these events, epigenetic constraints are removed so that embryonic stem cells develop, and pluripotency is promoted. On the other hand, some epigenetic patterns that are inherited from parents, like imprinted genes, are retained and not reprogrammed, while some imprints specific to parents are also established [10]. Specifically, primordial germ cells of embryos undergo epigenetic reprogramming, where pre-existing parental DNA methylation patterns and histone modifications are erased and reset, to impart *de novo* epigenetic landscapes that are unique to the offspring [11].

One of the mechanisms to retain parental epigenetic marks is through DNA elements called Intracisternal A particles (IAPs), which belong to the endogenous retrovirus (ERVs) family [12]. IAPs integrate into the mammalian genome and escape epigenetic reprogramming, and so do genes located near IAPs, even if they are in primordial germ cells [12]. For example, at embryonic day 13.5 of mice, the developing primordial germ cells are greatly hypomethylated but IAPs and nearby CpG islands retain methylation marks [12].

In genuine imprinted genes, monoallelic gene expression and parent-of-origin allelic transmission are observed, but only the latter is found in imprint-like genes, while monoallelic gene expression is not reported. An example of imprint-like genes is seen in germ cells, characterized by differential methylation patterns, where DNA methylation is perturbed by environmental conditions [13]. The epigenomes of the developing embryonic stem cells (ESCs) are changed when epigenetic information is transmitted through germ cells to future generations, affecting the dynamics of the epigenetic and transcriptomic landscapes of all somatic cells that are derived from the ESCs [7].

### 3.2. Transgenerational Epigenetic Inheritance in Disease

Studies also indicate transgenerational epigenetic inheritance stemming from human disease outbreaks like the Swedish and Dutch famines, where increased mortality risk from diabetes is observed in men whose grandfathers were exposed to famine, and in women whose grandmothers were exposed [14,15]. Epigenetic information carriers (unlike DNA) are highly dynamic and are often modulated by environmental conditions [16], suggesting that the environment experienced by parents may influence the phenotype of offspring via alterations to the gametic “epigenome” [17]. Studies of cell-state and transgenerational epigenetic inheritance have identified chromatin structure, DNA modifications, small RNAs, and prions as the main molecular carriers of epigenetic information [16].

### 3.3. Transgenerational Epigenetic Inheritance and Histone Modifications

Histone modifications are also implicated in transgenerational epigenetic inheritance [18,19]. In mammals, 1–10% of total histones are retained during spermatogenesis despite histone cores being replaced by protamines to accommodate DNA in the sperm head [7,20,21]. The histones that are retained in sperms regulate transcription in offspring [22]. Studies have reported that histones and their modifications are retained and inherited in mammals, including humans [23,24]. Human sperm histones retain histone H3 methylation alterations that correspond with fertility [25].

Research shows that retention of a specific set of histones in F3 generation controls lineage sperm of rats, which is likely linked to sperm-mediated transgenerational inheritance of illnesses following toxin exposure of previous generations [23]. The phenotype shows that the number of differential histone retention sites (DHRs) for histone H3 is high in F3, but low in F1 and F2 [18,24]. The studies explain that the phenotypes in F1 and F2 result from direct chemical exposure mechanisms, which is different from F3. The phenotype in F3 is caused by ESC reprogramming, which affects all downstream somatic cells and sperms, hence producing a different phenotype compared to F1 and F2 [18,24].

Studies on the female germline have reported that it mediates epigenetic transgenerational inheritance as well [26,27]. Overall, studies strongly indicate that when germlines are exposed to epigenome-altering environmental stimuli, the epigenomes of ESCs are perturbed, which impacts the epigenetic and transcriptomic landscapes of downstream somatic cell populations [7,28,29]. Since one of the roles of transgenerational epigenetic inheritance is proposed to be immune response to harmful environments, the next section focuses on the immune system and its relationship with epigenetic processes.

## 4. Overview of Immune System Development

**Figure 1:**
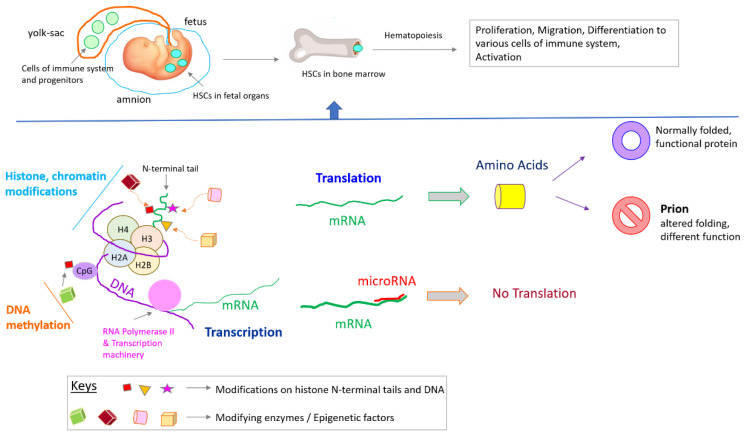
Epigenetic regulations of immune system development and activation. Abbreviations: HSCs—hematopoietic stem cells. H2A, H2B, H3, H4—core histones.

Development of the immune system occurs under tight temporal and spatial orchestration by various factors including epigenetics (Figure 1). The immune system comprises of diverse cell types including various categories of white blood cells, e.g., lymphocytes, neutrophils, monocytes, and mast cells, as well as molecules like antibodies, signaling proteins like cytokines, and complement proteins. Lymphocytes are classified into T-cells, B-cells, and NK (natural killer) cells, while monocytes migrate into tissues to become macrophages or dendritic cells. Generally, white blood cells originate from progenitor cells called hematopoietic stem cells (HSCs) in the bone marrow, but NK cells can also develop and mature in other lymphoid tissue [30,31,32,33,34,35]. The development of various cells of the immune system has been presented in detail by several studies; for that reason, those biological processes are not discussed in this review [30,36,37,38,39,40,41,42].

Developmental stages as early as four weeks post-conception show the presence of HSCs and immune cells in the human embryonic yolk sac [34,43]. During the early stages of development, certain tissues retain immune cells, e.g., macrophages, mast cells, NK cells, and innate lymphoid cell progenitors (ILCs), which originate from the yolk sac and/or differentiate from HSCs [34,43,44]. On the other hand, neutrophils appear only after bone marrow hematopoiesis begins [45]. Progenitors for B cells and T cells originate in the fetal liver at seven and eight weeks post-conception, respectively, after which T cell progenitors migrate to the thymus for maturation [43,46]. Overall, it has been observed that differentiation of various cells of the immune system leading to the final mature functional form is regulated temporally and spatially by distinct biological pathways and entities. In this direction, epigenetic regulation plays a crucial role in the development of the immune system [47,48,49].

## 5. Epigenetics in Immune System Development and Activation

During the first step of immune cell development, or hematopoiesis, an abundance of epigenetic signatures like H3K4me1 and *de novo* lineage-specific enhancers are observed, with the enhancer epigenetic profiles becoming more distinct during defined differentiation of monocytes to macrophages [50]. Epigenetic alterations in transcription factors, cytokines, and their modulators regulate gene expression profiles and the functions of developing memory T cells [51,52]. Interestingly, genome-wide analysis of histone marks like H3K4me3 and H3K427me3 in CD8+ memory T cells shows four epigenetic states—active, poised, bivalent, and repressed [51,53]. During immune system activation, H3K27ac and H3K4me3 are consistently enriched at the promoters and enhancers of the immune and stimulus-response genes [54,55].

In addition to histone modifications, another epigenetic process that regulates early development of the immune system including lymphoid tissue formation, survival and activation of immune cells, is DNA methylation at CpG islands. This epigenetic modification is generally implemented prenatally, but it is also established postnatally on a subset of genes including those regulating lymphocyte development and belonging to superfamilies of tumor necrosis factor receptors and cytokines [56]. DNA methylation-demethylation also regulates innate immune memory [57] through differentiation of myeloid cells, which elicit an innate immune response [58,59]. In innate immune cells, DNA demethylase TET2 oxidizes the RNA residue 5-methylcytosine (a modification that is heritable), governs gene regulation, and preserves cellular memory [60].

B-cell development is governed by the epigenetic regulation of HSCs, and the chromatin landscape remains dynamic during the process [51,61,62,63]. B-cell differentiation and activation are impacted by DNA methylation changes in gene bodies beyond CpG islands [64,65,66]. For B-cell function, certain transcription factors and their target genes undergo demethylation to get expressed in B cells, leading to immunoglobulin V(D)J recombination [51].

Another transcription factor that interacts with epigenetic factors is Blimp-1, which regulates the differentiation of B cells and T cells. Blimp-1 interacts with histone lysine demethylase LSD1 to repress transcription in mature B cells that promote the generation of antibody-secreting cells [67]. Blimp-1 recruits histone methyltransferase G9a and histone deacetylase HDAC2 to repress transcription that impacts the fate of effector CD8+ T cells [68,69]. Epigenetic processes also regulate the innate immune response to infection [70,71] and immune cell reprogramming during injury, repair, and resolution [72].

### Non-Coding RNA-Mediated Regulation of Epigenetics and the Immune System

The development and function of immune cells are further regulated by non-coding RNA (ncRNA), which are RNA that do not code for proteins. Interestingly, ncRNAs also regulate gene expression through various epigenetic mechanisms. Gene silencing by recruiting histone and DNA methyltransferase enzymes is the most reported epigenetic mechanism of ncRNAs [73]. ncRNAs promote the targeted recruitment of histone methyltransferases, to whom ncRNAs also provide scaffolds and structural complexes for assembly [73]. Categories of ncRNA include microRNA (miRNA), long non-coding RNA (lncRNA), small interfering RNA (siRNA), piwi-interacting RNA (piRNA), and small nucleolar RNA (snoRNA) [74].

miRNAs are about 22 nucleotides long and associate with active RNA-induced silencing complexes (RISCs) or microRNA ribonucleoprotein complexes (miRNPs) for gene silencing at the transcriptional and translational levels [75,76,77,78,79]. miRNA regulates DNA methylation and histone modifications by targeting their causative enzymes affecting various processes including gene expression and cell fate [80,81,82]. Through *de novo* DNA methylation, endogenous miRNA (miR-10a) can both transcriptionally enhance and downregulate (homeobox) transcription factor expression in human cancer cells [80]. miR-1 and miR-140 target HDAC4, which likely promotes cell differentiation during muscle and bone development, respectively [83,84,85]. miR-92b targets EZH2 to suppress breast cancer and promote autophagy, which is a critical process in the immune response [86].

The first step in immune cell development, i.e., hematopoiesis in the bone marrow, is also regulated by miRNAs [75]; they are expressed by various cells including hematopoietic precursors and their mature progeny [87]. Development of the immune function of macrophages, dendritic and mast cells is regulated by miRNA [88]. Additionally, miRNAs regulate the differentiation of B cells in bone marrow, and antibody responses [89]. For natural killer (NK) cells, miRNAs regulate maturation, homeostasis, and immune function [90].

miRNAs further regulate various stages of T-cell development and function. T-cell progenitors called thymocytes are stem cells that are produced in the bone marrow, which travel to the thymus through the bloodstream and differentiate into T cells. miRNA regulates the initial developmental stages of thymocytes, and also thymic epithelial cells, which are required for maturation and selection of thymocytes [91]. T-cell function during the immune response is also impacted by miRNA, which regulates antigen-presenting cells, accessory cells, and responding T cells. T-cell types like cytotoxic T cells, helper T cells, and regulatory T cells are also regulated by miRNA [92]. A network view of coding and noncoding RNA control of T-cell function has also been published [93].

As miRNAs regulate hematopoietic development, immune cell differentiation and activation, abnormalities of miRNAs are associated with autoimmune diseases like systemic lupus erythematosus, rheumatoid arthritis, systemic sclerosis, Sjogren’s syndrome, autoimmune thyroid diseases, type I diabetes, etc. [94,95,96].

siRNAs also cause post-transcriptional gene silencing by associating with RISCs and binding to mRNA due to sequence complementarity, following which RISCs degrade mRNA [97]. Transcriptionally repressive DNA and histone methylation marks, including H3K9me2, are mediated by siRNAs [98]. Small non-coding RNA also contribute to transgenerational epigenetic inheritance as seen in studies reporting their alterations in murine sperms [99,100,101,102]. In Drosophila, the absence of piRNA expression in piwi mutants corresponds to extensive decreases in H3K9ac, H3K4me2, and H3K4me3, indicating a role of piRNA in chromatin dynamics [103].

Like their smaller counterparts, long non-coding RNA (lncRNA) also regulate epigenetics. lncRNA span over 200 nucleotides and are not translated into proteins. One of their functions is epigenetic regulation by various mechanisms [104] like X-chromosome inactivation [105,106], intrachromosomal looping, and recruitment of DNA demethylase and chromatin-modifying enzymes [107]. lncRNAs are implicated in gene silencing by imprinting, which is an epigenetic process where the expression of only one allele of a gene occurs from either a maternal or paternal chromosome. lncRNA *AChE-AS* promotes histone methylation to suppress the acetylcholinesterase gene in hepatocellular carcinoma [108], while lnRNAs *ecCEBPA* and *Dali* inhibit DNA methylation to promote gene activation [109]. lncRNAs are also reported to occur as telomeric RNA (telRNA), which are transcribed from and localized to telomeres, suggesting a role in telomere-specific heterochromatin modifications [110,111].

## 6. Epigenetics at the Protein Level: Prions

Prions or proteinaceous infectious particles are proteins capable of shifting among more than one conformation, where at least one conformation is structurally templated for other similar proteins [112,113,114,115]. Hence, prions provide an additional mechanism for heritable information to be transmitted along the central dogma [90]. A single prion protein can attain complex stable and heritable activity states called prion variants or prion strains, containing the same polypeptide sequence, and prions show a non-Mendelian basis of inheritance [112,116,117,118,119]. Prions are found in eukaryotes, bacteria, viruses, and very recently, prion-like domains (PLDs) have been reported in Archaea, which indicates that prion-based inheritance is one of the most ancient epigenetic mechanisms [120].

Although some prions can be pathogenic, others may augment fitness under environmental stress because prions regulate physiology, resulting in higher phenotypic diversity [121,122,123]. Studies suggest that when proteins are damaged following stress and then cell division occurs, prions remain contained in mother cells to likely protect daughter cells from inheriting aggregates of damaged proteins [124]. Thus, prions undergo asymmetric inheritance during cell division to maintain population fitness, which likely impacts cellular plasticity [121].

### 6.1. Role of Prions in Gene Expression Related to the Immune System

Studies have reported prion-like characteristics for two activators of antiviral immune responses in mammals called MAVS (mitochondrial antiviral signaling protein) and ASC (Apoptosis-associated speck-like protein containing a CARD; CARD—C-terminal caspase-recruitment domain) [119,125,126]. Those studies further report that MAVS interacts with a sensor of viral infection and forms bulky aggregates, resulting in interferon expression through the activation of IRF3 (Interferon Regulatory Factor 3), which is a transcription factor. Tia-1 or T-cell inducer antigen 1 also shows prion-like properties, whose roles include mRNA binding and formation of stress granules and amyloid aggregates [119,127,128]. Studies on prion protein (PrP) indicate its upregulation in CD8+ cells, which proliferate in a homeostatic manner after introduction into murine models of lymphopenia [129]. The observation was inferred from gene expression microarray analysis, and it supports the role of PrP in lymphoid repopulation [129,130].

Likewise, PrP upregulation and surface expression are detected in memory differentiation [131]. PrP knockout in HSCs decreases their self-renewal [132], while prion protein is one of the surface markers detected in freshly isolated murine HSCs, which contributes to the plasticity of their surface phenotype [133]. In peptide-challenged PrP−/− murine models, mitosis decreases upon the introduction of PrP+/+ TCR tg T cells, where TCR tg denotes ‘T-cell receptor transgenic’ [134]. The same study also showed that the absence of PrP in dendritic cells significantly lowers the proliferation of interacting T cells. Collectively, studies have established that PrPc, which is the cellular isoform of PrP, contributes to T-cell proliferation and differentiation [130], and immunological quiescence [135]. Furthermore, PrPc plays a role in regulating HSC counts during aging, myeloid progenitor fates of HSCs, and offers protection to myeloid progenitors from irradiation [136]. Studies have further indicated that PrPc likely regulates the maturation and exit of certain cell lineages from the bone marrow, rather than major perturbations of immune cell functions, under conventional growth in the absence of stressors [137].

**Figure 2:**
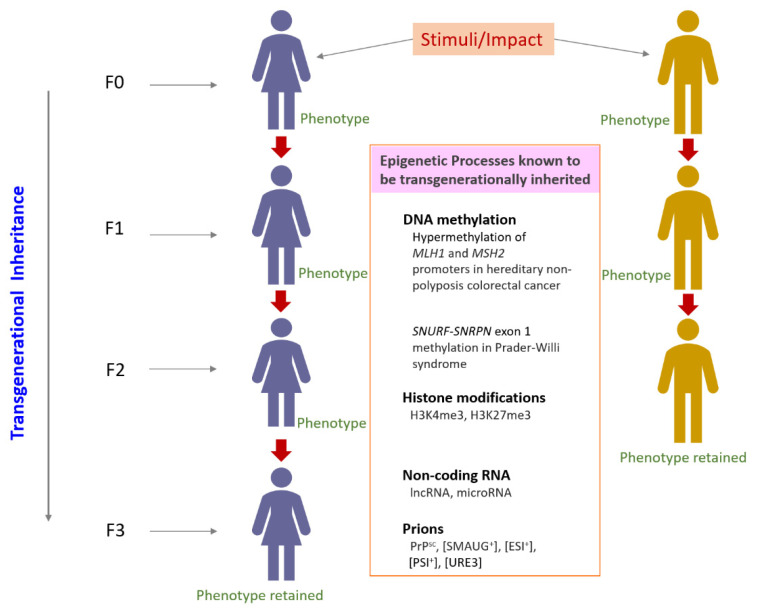
Transgenerational epigenetic inheritance and some phenomena reported in humans in the context of disease. MLH1 [138], MSH2 [139], SNURF-SNRPN [140], H3K4me3 and H3K27me3 [23,141,142].

Overall, prions, DNA methylation, histone modifications, and non-coding RNA have been reported across species and human diseases in the context of transgenerational epigenetic inheritance (Figure 2).

### 6.2. Prions and Epigenetic Inheritance

Epigenetic inheritance of conformationally altered or misfolded PrP causes diseases including fatal familial insomnia, and a rise in stress conditions increases the rate of occurrence of misfolding, and hence, epigenetic changes [143,144]. The transition of naive progenitor chromatin to B-cell lineage-committed chromatin is facilitated by the prion-like domain (PLD) contained in the C-terminal domain (CTD) of transcription factor EBF1 [145]. The process involves the binding of EBF1 to the progenitor cell chromatin followed by EBF1-CTD targeting the recruitment of chromatin remodeler BRG1 [145].

Prions play a crucial role in neurodegenerative diseases, where normal PrPc is converted into a misfolded scrapie isoform called PrPSc pathogenic, which provides a seeding mechanism for the propagation of pathogenic prions [146]. In neuronal cells, the above conversion leads to molecular and functional alterations that impact synaptic plasticity. Cells with PrPc depletion and prion infection show defects in two important signaling pathways, namely Notch and Eph, which are crucial for development and axon migration, respectively [146]. The defects are ameliorated by inhibiting histone deacetylase, hence, Notch and Eph pathways are epigenetically regulated by prion [146]. In other words, epigenetic regulations sustain changes associated with loss-of-function phenotypes regarding Notch and Eph signaling that result from pathogenic prions [146].

### 6.3. Prions and Transgenerational Epigenetic Inheritance

The epigenetic inheritance of prions shows transgenerational stability as prions are transmitted to mitotic progeny [147,148,149,150]. Most prion states replicate and are inherited with the help of chaperones, which directly connect their inheritance to environmental stress [147]. In prions, often biased glutamine/asparagine-rich sequences are arranged in domains that regulate the assemblage of multiple self-templating polymorphs, or prion strains [147]. Nuances in prion domain sequences result in strong transmission barriers among species [147]. Dramatic phenotypic changes can result from conformational switches of prions, and such traits are inherited in daughter cells as a kind of extrachromosomal epigenetic ‘memory’ [112,144]. Although very stable, prion-based inheritance is also readily reversed, which indicates that prion-mediated changes are likely needed to fit in with erratic environments [112,151,152].

In yeast studies, epigenetic elements [PSI+] and [URE3] are prion forms of Sup35p and Ure2p, respectively [153]. Unlike genetic traits, the inheritance of these elements can be permanently removed by environmental stress; while they are inherited by all meiotic progeny obtained from genetic crosses, unlike genetic traits that are only inherited in 50% of the daughter cells [112]. A recent study reports a role of prion [SMAUG+] in the rewiring of post-transcriptional gene expression that promotes robust mitotic development [154]. Interestingly, prion [SMAUG+] is formed by non-amyloid self-assembly of an RNA-binding protein which causes heritable activation of protein function, and the self-assembly is conserved in humans [154]. Hence, the study indicates that such non-amyloid self-assembly can induce adaptive gene expression processes, which are likely inherited across lengthy biological timelines or transgenerationally [154].

Research on prions has further shown that active chromatin states can also undergo transgenerational inheritance [155]. It is a new observation in the field of transgenerational inheritance because mostly repressed chromatin was known to be inherited in the past. The study reports that prion [ESI+] results from cell cycle arrest-mediated transient phosphorylation of Snt1, which is a scaffold for the epigenetic regulator Set3C histone deacetylase [155]. Next, [ESI+] modulates Snt1 and the Set3C complex to epigenetically switch sub-telomeric domains from transcription repression to activation states [155]. As a result, resistance to environmental stress, and phenotypes for an adaptive benefit, is achieved [155]. From a mechanistic perspective, cells containing [ESI+] show increased histone H4 acetylation, which is a transcriptional activation mark, leading to the elevated abundance of about 1000 transcripts and increased association of RNA polymerase II [155]. It will be interesting to discover how the various epigenetic factors associated with transgenerational inheritance and the immune system affect immune-related diseases.

## 7. Immune-Related Pathologies Involving Transgenerational Epigenetic Inheritance

One of the causes of immune-related pathologies is transgenerational epigenetic inheritance induced by chemical exposure [7,156]. A study showed that fungicide exposure to F0 generation alters DNA methylation in sperms, leading to disrupted transcriptome in various tissues, causing immune abnormalities in generations F1 to F4 [157]. Fetal exposure to alcohol detrimentally affects the immune system through transgenerational inheritance of epigenetic modifications of Ifn-γ, a key immune gene [158]. Transgenerational transmission of alcohol-induced epigenetic modifications in stem cells affects brain development and memory dysfunction in successive generations [159]. Hence, knowledge of transgenerational epigenetic inheritance is beneficial to address substance abuse.

Neurodegenerative disorders like Alzheimer’s disease (AD) are also at the crossroads of transgenerational epigenetic inheritance [54,160,161] and immune response [162]. Transgenerational events in AD are mediated by amyloids that are similar to prions, which are protein-level epigenetic factors [161]. As potential remedies, transgenerational benefits of choline supplementation in maternal diets [160] and epigenetic factors like DNA methylation, histone modifications, and ncRNAs are under focus [160]. Some allergic immune responses are also linked to transgenerational epigenetic inheritance [163].

The process is further implicated in pathological outcomes of neurodevelopmental disorders, as seen in epidemiological studies [164]. The scope of the field further extends to psychiatry as a study on rats shows that mental stress to ancestors causes transgenerational inheritance of alterations in immune response and metabolism, which can aid in discovering biomarkers for improved diagnosis and control of mental health disorders [165].

## 8. Epigenetic Inheritance and the Immune System in the Context of Aging

Epigenetic regulations not only impact the developmental and maturation of cells of the immune system, but they also impact the aging of the immune system [166]. Analysis of the methylome of immune cells like CD4+ T cells from newborns and centenarians reveals similar DNA methylation profiles like other tissues while aging, hypomethylation of DNA at a global level, and increased variability of DNA methylation [167]. Other studies on naïve CD4+ T cells have also reported hypomethylated sites with increasing age, and active enhancers enriched in histone modifications like H3K27Ac and H3K4me1 [168]. The observations indicate that T-cell epigenetic landscapes progressively shift toward pro-inflammation and T-cell activation with age, likely leading to autoimmunity [166,168].

Populations with high old-age life expectancy, like Nicoyans from Costa Rica, have a significantly increased abundance of predicted CD8+ T naïve cells and reduced counts of estimated CD8+ T memory cells in comparison to non-Nicoyans [169]. The observation suggests a younger profile of immune cells [166,169]. Another epigenetic feature of Nicoyans is lower variations in DNA methylation patterns in contrast to other populations [169]. In the context of B cells, the ability of HSCs to differentiate gets reduced with age, and the process is regulated by epigenetic factors [166]. Studies have shown that epigenetic dysregulation causes anomalous transcriptomic profiles in aged murine HSCs [166,170].

## 9. Methods to Study Epigenetics and, Hence, Transgenerational Epigenetic Inheritance

Studies on most conventional model organisms, including yeast, Drosophila, zebrafish, rodents, and humans have proved the existence of transgenerational epigenetic inheritance mechanisms. With the development of high-throughput technology to study gene expression and the chromatin landscape, structure, and dynamics, it is becoming more convenient to study epigenetic mechanisms. Advances in bioinformatics techniques and algorithm development have made genome-wide analysis and comparisons across multiple subjects in a cohort, or multiple species with different genomes, very quick and convenient. The techniques have been evolved to study not only cells in bulk but also to trace cell lineages in conducting single-cell analysis.

In this direction, single-cell RNA sequencing and single-cell ATAC-seq (assay for transposase-accessible chromatin using sequencing) are used to study transcriptome and open/closed chromatin, respectively, on a genome-wide scale. Several studies on the immune system are using these techniques for high throughput and resolution. Heterogeneity of immune cells, development of distinct subpopulations from progenitor cells, and subpopulation-specific gene expression profiles can be studied using single-cell RNA sequencing [171]. ATAC-seq has enabled the discovery of various aspects of the immune system, including heterogeneity, epigenomic signatures, fate, and regulatory pathways of T cells [172].

The genome-wide distribution of protein-DNA interactions, transcription factors, and histone modifications can be analyzed using techniques like ChIP-seq (chromatin immunoprecipitation with sequencing), CUT&TAG (cleavage under targets and tagmentation [173]), etc. Both ChIP-seq and CUT&TAG have their own advantages and disadvantages, and the field is constantly evolving to improve upon existing technology due to high demand. ChIP-seq has been extensively used to study the immune system, including mapping of transcription factors, critical effectors, and epigenetic modifications that regulate immune response and specific developmental steps of various immune cells [174]. Deep learning methods to study immune cell differentiation in silico [175] are also being developed that go in conjunction with ChIP-seq results. Using single-cell CUT&TAG, a study identified an immune response based on microglial cell activation in specific cell populations [176].

To study the genome-wide chromatin architecture, Hi-C has been developed, which detects changes in chromatin organization based on the impact of stimuli, and chromatin organization patterns that correspond to various developmental and disease stages. Hi-C is also being used to study immune-mediated diseases [177]. Technological advancement in terms of sensitivity, resolution, throughput, and sample requirement will make the study of epigenetic regulations of the immune system more convenient.

## 10. Clinical Trial on Transgenerational Intervention

Although some epigenetic modifiers are targeted by therapies against cancer and other diseases, with more epigenetic factors undergoing clinical trials, therapies focusing on transgenerational epigenetic inheritance mechanisms are yet to become popular. As a stepping stone in this direction, one clinical trial from the USA’s University of North Carolina and National Institute of Diabetes and Digestive and Kidney Diseases (NIDDK) focuses on transgenerational e-intervention for gestational diabetes (clinicaltrials.gov/ct2/show/NCT01931280 (accessed on 1 April 2021)). The primary outcome of the study is the change in glycosylated hemoglobin.

This study is interesting as it measures glycosylation to address a transgenerational question during gestational diabetes, which involves low-grade systemic inflammation with a pro-inflammatory immune system response. Glycosylation is an important epigenetic modification of histones [178,179]. This is because nucleocytoplasmic glycosylation or O-GlcNAc modification has emerged as a novel epigenetic factor that regulates gene expression through histone modifications, histone-modifying enzymes, RNA polymerase II, etc. [179]. Furthermore, histone glycation from persistent hyperglycemia impacts electrostatic interactions, causing histone-histone and histone-DNA crosslinks in chromatin, which alters chromatin dynamics and results in cancer [178]. Hence, glycosylation is a crucial epigenetic phenomenon that is focused on in the above clinical trial.

With further discoveries on the impacts of transgenerational epigenetic inheritance, glycosylation and other epigenetic modifications will attract more focus from a therapeutic and clinical trial perspective.

## 11. Conclusions

Building on current studies and technological advances, further exploration of the molecular mechanisms behind transgenerational inheritance of specific epigenetic factors and their pathological outcomes will be beneficial from discovery and therapeutic perspectives. Identification of new targets at various levels of epigenetic modifications like DNA methylation, histone modifications, ncRNA, and prions will provide new insights into how these factors can regulate transgenerational inheritance and impact diseases including immune disorders.

A practical application of knowledge on epigenetics and the immune system is seen in several clinical trials, which can show new directions as to how epigenetic modulation can be used to treat immune disorders. For example, a clinical trial is investigating epigenetics regarding stem cells and trained innate immunity in patients with atherosclerosis, which is an immune disease (clinicaltrials.gov/ct2/show/NCT03172507 (accessed on 1 April 2021)). Lupus is an autoimmune disease where DNA methylation is employed in one of its clinical trials (clinicaltrials.gov/ct2/show/NCT04648059 (accessed on 1 April 2021)). DNA methylation sequencing and RNA-seq are some of the tools used in a clinical trial against immune-mediated eye diseases (clinicaltrials.gov/ct2/show/NCT00647439 (accessed on 1 April 2021)) and asthma (clinicaltrials.gov/ct2/show/NCT01382836 (accessed on 1 April 2021)). Histone deacetylase inhibitors (HDACi) are used in clinical trials involving graft versus host disease (clinicaltrials.gov/ct2/show/NCT01111526 (accessed on 1 April 2021)), and immune checkpoint blockade in cancers (clinicaltrials.gov/ct2/show/NCT03233724 (accessed on 1 April 2021)). Some clinical trials on autoimmune diseases like rheumatoid arthritis and lupus focus on pregnancy-induced epigenetic changes regarding microRNAs (clinicaltrials.gov/ct2/show/NCT02350491 (accessed on 1 April 2021)).

Based on the above clinical trials, studies can be designed to specifically address immune profiles and epigenetic landscapes in diseases that are transgenerationally inherited. During the ongoing COVID-19 pandemic, epigenetic profiles of patients are under focus due to chromatin landscape changes of ACE2 and other histone modifications [180,181,182,183,184]. Although it is too early to comment on the transmission of the COVID-19 infection or its impact on epigenetic and immune profiles across generations, studies indicate a possibility [185].

The transgenerational inheritance of immune response is important beyond biomedical research as it has been linked to immune priming, which is a memory-like event occurring due to any sub-lethal exposure that prepares the immune system to combat a future lethal exposure [186]. In this direction, findings on farm animals [187], birds [188], plants [189,190], microbes [191], and invertebrates like Artemia [192,193] and Lepidoptera [194] link transgenerational epigenetic inheritance to immunity. Hence, the process impacts agriculture, the environment, and ecology. One study showed that when Artemia is exposed to pathogenic bacteria, then three subsequent generations of progenies show altered expression of major immune-related genes, with stochastic patterns of H4 acetylation and H3K4me3 [192]. It is only a matter of time before we will discover similar well-defined mechanisms in vertebrates.

Overall, the significance of transgenerational epigenetic inheritance is already established [5,195,196,197] with a major outcome being adaptability to stress; hence, why its intricate relationship with immune system development and activation must be focused upon.

## Data Availability

Not applicable.
